# Cases of Peritoneal Section for the Extirpation of Diseased Ovaria, by the Large Incision from Sternum to Pubes, Successfully Treated

**Published:** 1843-10

**Authors:** 


### ART. X.

*Cases of Peritoneal Section for the Extirpation of Diseased Ovaria, by the large incision from Sternum to Pubes, successfully treated.* By CHARLES CLAY, Member of the Royal College of Physicians, &c.—*London*, 1842. 4to, pp. 18.

DR. CLAY's work is a reprint of several papers which originally appeared in the pages of the Medical Times. It contains the particulars of five cases in which gastrotomy was performed by Dr. Clay, and in three of these cases the patients recovered. This measure of success, backed by the evidence of statistics, which show, according to Dr. Clay, that twelve out of thirteen patients who underwent the operation ultimately did well, induces him to recommend it as "a perfectly legitimate and more than ordinarily successful capital operation." But, not merely does he conceive the propriety of extirpation of the ovaria to be fully established by these cases, but he regards them as forming the commencement of a new era in operative surgery. Nor can we dispute the correctness of this opinion if he should ever put his own intentions in practice, for he tells us: "I feel I should be justified in making extensive incisions into the abdominal cavity, for other objects than the one here related. I would ask, what should prevent the spleen from being extirpated when diseased? Or fatty tumours of the omentum? Or tumours of the fundus uteri?"

We believe, indeed, that the good sense of mankind will answer these questions, and that Dr. Clay's offer to extirpate some offending viscus will be met by most people with the "*minime tangetur*" with which the hero of Slawkenbergius's tale declines all manual interference with his proboscis. But let that pass. Dr. Clay, though in one place he asserts that this operation, "when compared with the results of lithotomy, lithotrity, and even amputation, stands in a far more favorable position;" yet seems to have some lurking misgivings on the point, since in another place he talks of repeating the operation until he has ascertained its real value. We give the whole passage in Dr. Clay's own words, the rather that it shows at once his estimate of his own merits as well as of the operation. It would not have occurred to us to place the author in the temple of fame by the side of Harvey, Jenner, and Cullen, even if his future efforts should be met by the profession in the way he anticipates.

"It is my intention to persevere in this operation until I have produced sufficient evidence to establish a legitimate operation of British (as it is already of foreign) surgery; or until I discover enough to condemn it. In accomplishing this, I shall find much greater difficulty in overcoming the feeling of reluctance generally manifested by the profession towards any new and bold step (though supported by numerous proofs), than I should in meeting with condemnation for the unfortunate result of some one or two isolated cases, which in no way affects the principle of the operation, but affords arguments to those who are prejudiced against its being adopted as a legitimate means of relief. In this, however, I am not singular: Harvey met with the most virulent opposition, for many years, to doctrines since acknowledged to be founded on truth. Jenner's discoveries were as strongly opposed, and as firmly admitted subsequently. Cullen's views were attempted to be driven out of the pale of the professional inquirer by the numerous followers of Brown." (p. 16.)

Before we proceed to consider the statistical grounds on which Dr. Clay defends the operation of gastrotomy in ovarian disease, we must briefly narrate the particulars of those cases in which he was himself the operator. In the management of them great credit is due to him for his sedulous attention to some minor points such as the temperature of the room, &c., the neglect of which may probably have contributed to the fatal result of other cases. The minute details too that he has given of the history of some of the patients and of their progress after the operation are highly praiseworthy, and induce us to acquit him of wilful disingenuousness in his erroneous statistics, though we cannot but consider him guilty of most culpable negligence in not investigating the subject with greater care before proceeding to dogmatize upon it, in the "Sir Oracle" tone which prevades his pamphlet.

Dr. Clay's *first case* was that of a married woman, forty-six years of age, mother of eight children. Three years before coming under his care she had noticed her abdomen begin to enlarge, and at first she thought herself pregnant, but gave up this opinion on finding the menses occur with regularity notwithstanding the progressive increase in the size of the abdomen. The disease was unattended with pain, the sensation being merely that of weight and incumbrance which she felt on the right earlier than on the left side. After consulting various medical men, none of whom gave the patient any hopes of relief, she applied on June 10th, 1842, to Dr. Clay, her abdomen being then as much distended as it is usually at the ninth month of pregnancy. The pelvis was found to be filled with a large immoveable tumour, which appeared distinct from the uterus; and the uterus itself was lifted up higher out of the pelvis than usual, the os uteri resting against the upper and inner portion of the symphysis pubis. Dr. Radford, who saw the case, was of opinion that the tumour was formed by a greatly enlarged ovary, most probably the right; that it was free from adhesions beyond its principal attachment; and that with it there coexisted a small quantity of fluid in the peritoneal cavity. Trial was made of iodine, without any local benefit, while the patient's health seemed to suffer from its use; and the chances of recovery afforded by the operation as well as the hazard attending it having been explained to the patient she resolved to submit to it, and accordingly it was performed on September 12th, 1842. An incision was made along the linea alba from the sternum to the pubes, but leaving the umbilicus a little to the left. The peritoneum was then laid open to the same extent, the ovarian tumour was found free from any but three or four very slight adhesions, and one very firm connexion with the omentum. It sprang from the right side and was attached by a pedicle three inches in breadth to the broad ligament. A ligature being passed around the pedicle it was divided, but several vessels continuing to bleed, it was found necessary to tie each of them. From making the incision through the integuments to the removal of the tumour out of the abdomen about twelve minutes and a half elapsed, and about fourteen ounces of blood were lost, and the wound having been closed by the interrupted suture, and by strips of adhesive plaster and a bandage, the patient was replaced in bed about forty-five minutes from the commencement of the operation. The tumour weighed seventeen pounds five ounces, its largest circumference was three feet eight inches, its form rather oval. It consisted of one large and several small sacs containing a glutinous matter, and of a solid portion made up of very small cells filled some with pus, some with a brain-like substance, the intestines between them being fibrous and cartilaginous. There were besides about six pints and a half of fluid in the peritoneal cavity.

The pulse, which was 70 before the operation, continued for the next twenty-four hours at about 90, and rose but once above 100. It was soft and compressible, except at the eighth and nineteenth and a half hour after the operation; and at each of these times the patient was bled, first to ℥xiv. then to ℥viij. Enemata were given to act on the bowels; morphia and hyoscyamus to procure sleep: some vomiting and tympanitis were the only symptoms at all troublesome. The wound was not dressed until the fifth day after the operation, when it was found to have united, with the exception of half an inch at the umbilicus and half an inch where the ligature passed out of the abdomen. The absence of all grave symptoms is the chief point in the subsequent history of the patient, who left her bed during part of the day on September 28th, and returned to her household duties on October 3d, the twenty-third day after the operation.

Case ii. A woman aged fifty-seven, mother of nine children, was tapped for ascites on September 27th, 1842, when twenty-five pounds and a half of fluid were removed. A tumour was then detected in the left iliac region; circular, rather flattened, moveable, but connected with the abdominal parietes for the space of about two inches square. The patient had not been aware of the existence of any tumour ten months before; but within that period the fluid had begun to collect in the abdomen. It does not appear that she suffered except from the unwieldiness of her abdomen, and from pains about the umbilicus. Her general health had been good, but she had grown much emaciated within the last six or eight months. On October 7th, 1842, the operation was performed, and Dr. Clay was surprised to meet with adhesions in every direction; he having expected to find the tumour free, except in the vicinity of the umbilicus. The adhesions were divided by the scalpel; a strong double ligature was passed through the central expansion of the pedicle and tied both ways, a proceeding which was found adequate to secure the vessels without tying them separately, as in the former case. The operation was completed in about ten minutes, and the patient was replaced in bed in twenty-five minutes, not having lost more than two ounces of blood.

The tumour weighed five pounds: it was composed of a white tough membranous bag, capable of holding about four pints, and of a flattened round solid mass, the cells of which contained various matters from the consistence of pus to that of cerate, and of considerable variety in colour. This patient had not a single bad symptom. The wound was dressed on the fourth day for the first time. On the ninth day she was allowed to sit up, and on the 16th was considered as cured.

The *third* successful case is that of a woman named Hannah Edge, aged thirty-nine, the mother of three children. She first noticed a considerable enlargement of the lower part of the abdomen, on the right side, seven years previously. Four years before coming under Dr. Clay's care she was delivered of her third child; but after its birth her abdomen continued nearly as large as that of a person at the full period of pregnancy. She had been tapped four times; and on November 3d, 1842, the operation was repeated, when thirty pounds of dark glairy fluid were removed, and, on introducing a longer trocar, other thirty pounds of bright limpid fluid were drawn off. It was determined to perform the operation of extirpation; and this was accordingly done on November 8th. Owing to some oversight of the printers, the particulars of the operation and the description of the tumour are omitted. It appears, however, that the adhesions were numerous, but that the patient nevertheless did well; and from the detailed account given of her progress from the termination of the third day, her recovery appears to have been more rapid than that of either of the other patients, since on the eighth day she sat up, and on the twelfth was so well as to require only occasional attendance.

In neither of the two fatal cases were the histories of the patients such as to induce Dr. Clay to recommend the operation, though he felt justified in performing it in consequence of their urgent solicitations.

The first case was that of a woman aged forty-seven, who, though married eight years, had never had any children. She had first noticed a tumour in her left iliac region seven years before applying to Dr. Clay, but for the first three years it had enlarged very slowly. Its subsequent increase in size had been rapid; and at the time when seen by Dr. Clay her abdomen measured forty-five inches in circumference at the umbilicus. Her sufferings had been considerable; and two years before she had been tapped, but only two pints of bloody fluid escaped. It was her earnest wish to undergo the operation for the removal of the tumour: an incision was accordingly made, but the growth was found so universally adherent that all attempts to remove it were considered unwarrantable, and the wound was closed immediately. The case went on tolerably favorably until the fifth day, when the patient was seized with pain of the left leg, which had much the appearance of a limb affected with phlegmasia dolens. Some relief was afforded by warm fomentations; but the pulse sank very rapidly, and the patient died on the morning of the sixth day, in less than twelve hours from the commencement of the attack. The attack is attributed by Dr. Clay to the woman's husband having given her some gin and garlic on the morning of the fifth day after the operation.

Few particulars only are recorded of the patient who forms the subject of the second fatal case. She was forty-five years old; the abdomen had the size which it presents in a person at the eighth month of uterogestation. The abdominal tumour had a hard and lobulated feel; no fluctuation could be anywhere detected in it, but it seemed not to have the slightest peritoneal attachment. An incision was made into the abdomen; the tumour was found free from peritoneal attachments but adherent to both fallopian tubes, and involving a considerable portion of the uterus. Ligatures were applied round the fallopian and uterine attachments, and the tumour was removed. Before this was accomplished, however, the patient had fainted; the ligatures were insufficient to prevent hemorrhage from the divided vessels; the patient fell into a state of syncope, and died within an hour and a half from the removal of the tumour.

Before we proceed to the examination of Dr. Clay's statistics it will perhaps be but right to narrate the particulars of a case in which Mr. Walne, encouraged by Dr. Clay's success, removed a large ovarian tumour by an operation similar to that above described, and in which the patient recovered. She was a married woman, fifty-eight years of age, who had had five living children and several miscarriages, and had ceased to menstruate at the age of fifty-four. Her health had been good; but for more than two years she had been gradually getting larger in the abdomen, in which was a circumscribed fluctuating tumour, but without any signs of general dropsy. She measured thirty-seven and a half inches round, and seventeen inches from scrobiculus cordis to pubes. It was determined to operate; and the operation was done in a room heated to 70°, at 4 P.M. on Nov. 6th, 1842. The incision was thirteen inches long; made in the linea alba, but avoiding the umbilicus. The right ovary was found to be the one affected; a double ligature was placed by means of a needle around the pedicle, which was then divided and the tumour, weighing sixteen pounds, was removed. A large artery in the pedicle was now tied; but as oozing of blood from the pedicle continued, a ligature of stay silk was placed around its circumference. The wound was brought together by a dozen interrupted sutures, and a broad bandage was applied round the abdomen. The pulse after the operation was 76, and the patient was pale and cold. No unfavorable symptom occurred for the first twenty-four hours: almost total abstinence from food and drink was observed. The second night was less good; considerable fever and restlessness came on, and continued during the following day, with occasional vomiting and eructation. Great relief was afforded by an enema of warm water, and an anodyne was followed by some hours of comfortable rest. On the 9th the patient passed urine of her own accord, and was going on well; on the 10th there was some restlessness, with vomiting and griping, which were more distressing on the 11th, with constant nausea and frequent hiccup. These were checked by anodynes, though they did not altogether cease, and returned occasionally; the hiccup not finally disappearing till about the 20th. On the 23d she sat up several hours; on the 27th the ligature which had been placed round the artery of the pedicle was removed; on the 29th the wound was healed, except a small opening at the bottom near the pubes. The greatest circumference of the tumour was two feet ten inches and three quarters: it contained fluid in one or more cysts; a portion, the size of two fists, had a scirrhous hardness, but Mr. Walne did not choose to spoil the preparation by cutting into it.

Including this first case of Mr. Walne's, Dr. Clay reckons thirteen in which the operation has been performed, of which only one terminated fatally; while five of the ten patients operated on by the small incision, as practised by Mr. Jeaffreson, died. His own fatal cases he places in a different category, as being instances in which an attempt was made to remove anomalous malignant uterine tumours. To this, however, we object, as by no means a fair mode of estimating the results of the operation. It is clear that Dr. Clay thought himself warranted in performing the operation in these cases; that he anticipated from it a prolongation of the patients' lives; but that in this he was mistaken, and that their death was greatly expedited by his interference; and, further, we have no doubt but that if he had been at all aware of the real nature of the diseased growths he would not have attempted their removal. This very impossibility of knowing beforehand the exact condition of the organs which it is proposed to extirpate, (of which Martini's case, and that related by the anonymous writer in Froriep's Notizen, are melancholy examples,) forms in our opinion one of the strongest arguments against the operation; and will always do so until we are furnished with some unerring means of distinguishing between simple encysted ovarian tumours, and cases in which the ovarian cysts are associated with other diseases of those organs. To leave out those cases in forming an estimate of the operation seems to us as unjust as it would be, in calculating the results of lithotomy, to omit all instances in which the patient's danger had been aggravated by disease of the bladder or kidneys.

Dr. Clay's statistical account of cases in which dropsical or enlarged ovaria were extirpated by the large incision, is as follows:

| | Successful. | | Fatal. |
|---|---|---|---|
| L'Aumonier, France . . | 1 | ... | 0 |
| Dr. Smith, Connecticut . . | 1 | ... | 0 |
| Dr. Macdonald, Kentucky . | 3 | ... | 0 |
| Mr. Lizars, Scotland . . | 3 | ... | 1 |
| Dr. Clay, Manchester . | 3 | ... | 0 |
| Mr. Walne, London . . | 1 | ... | 0 |
| | 12 | ... | 1 |

This statement is lamentably and unpardonably incorrect as a representation of the actual state of experience in this matter. On this account we must beg our readers, in forming their conclusions respecting the operation, to substitute the following tables, of which the first displays those cases in which the diseased ovarium was actually extirpated; the second, those in which either no tumour existed, or in which insurmountable obstacles prevented the completion of the operation.

Table I.

| | Successful cases. | | Fatal cases. |
|---|---|---|---|
| Dr. Macdowell . . | 3 | ... | 1 |
| Mr. Lizars . . . | 1 | ... | 1 |
| Dr. A. G. Smith, of Danville, Kentucky | 1 | ... | 0 |
| Dr. Quittenbaum . . | 1 | ... | 0 |
| Dr. Rogers . . . | 1 | ... | 0 |
| Dr. Ritter . . . | 1 | ... | 0 |
| Dr. Chrysmar . . . | 1 | ... | 2 |
| Dr. Granville . . | 0 | ... | 1 |
| Dr. Clay . . . | 3 | ... | 1 |
| Mr. Walne . . | 1 | ... | 0 |
| | 13 | ... | 6 |

TABLE II.

| | Patients survived. | | Patients died. |
|---|---|---|---|
| Dr. Macdowell | 1 | ... | 0 |
| Mr. Lizars | 2 | ... | 0 |
| Dr. Granville | 1 | ... | 0 |
| Prof. Dieffenbach | 1 | ... | 0 |
| Dr. Martini | 0 | ... | 1 |
| Anonymous writer in Froriep's Notizen | 0 | ... | 1 |
| Dr. Clay | 0 | ... | 1 |
| | 5 | ... | 3 |

It appears then that, instead of the operation being successful in twelve out of thirteen cases, nine out of twenty-seven persons, or exactly one in three of those who submitted to it, died; and that five of the eighteen survivors had hazarded their lives and undergone much suffering to no purpose. But an examination, even of the successful cases, will show that the laudatory terms in which Dr. Clay speaks of this operation are by no means borne out by fact.

We have rejected L'Aumonier's case, which Dr. Clay enumerates among the instances in which the operation by the large incision was performed with success, because it has in reality no bearing whatever on the subject. With just as much propriety might he appeal to the tale told by Wierus in his book *De præstigiis*, of the sow-gelder, who, scandalized at the light life and conversation of his daughter, removed her ovaries by way of mending her morals. L'Aumonier's case is, to the best of our belief, wholly unprecedented in the annals of operative surgery; a bad pre-eminence which we trust it may long retain. The patient was a young woman, twenty-one years old, who came under M. L'Aumonier's care between six and seven weeks after delivery. She was then much emaciated. suffering from febrile symptoms, with a purulent discharge from the vagina, increased by pressure on the hypogastric region, which was tense and painful, and in which a hard tumour could be felt. The patient's strength had been further exhausted by obstinate diarrhea, and her powers of body were failing. M. L'Aumonier made an incision four inches long in a direction corresponding to the inferior edge of the *obliquus externus* muscle; and came upon a large round tumour, with which the ovary was connected. This tumour on being punctured gave issue to a pint of dark fetid pus, and it was now ascertained that the cavity in which it had collected was that of the Fallopian tube. With the cavity of the Fallopian tube the ovary communicated; an abscess having formed in its substance and burst into the tube. The ovary was not much enlarged, nor does there exist any evidence of its having been the seat of any other morbid process than inflammation terminating in suppuration. M. L'Aumonier, however, feeling "bien certain que les désordres de son organisation étoient irréparables," tore away the adhesions between the fimbriæ of the Fallopian tube and the ovary, which he removed. For the first six hours the patient was extremely exhausted and had slight convulsions; and during the whole of the next day she was scarcely able to speak. Though in a very precarious state she went on tolerably well from the third day till the sixteenth, when violent cerebral symptoms came on; ceasing, however, on the appearance of the menses, and at the date of the last report, forty-seven days after the operation, she might be considered quite recovered.* Now all that we can say to this is, that M. L'Aumonier and his patient were very lucky, but, surely if Dr. Clay had ever read the case he would not have thought of classing it among those which form the subject of his paper.

Dr. Smith of Connecticut's† case, was not, as Dr. Clay alleges, a case of operation by the large incision, but one in which the tumour was punctured and then drawn out through a small aperture in the abdomen, as in the mode practised by Mr. Jeaffreson. It is true that the incision in this case was four inches long instead of an inch and a half, though the operation was in other respects conducted according to Mr. Jeaffreson's plan. The case, however, terminated successfully, and is therefore classed by Dr. Clay among those operated on by the large incision, though the opening into the abdomen was only four inches long, while Dr. Stilling's‡ case, in which the incision was six inches long, is referred to the category of those operated on according to Mr. Jeaffreson's method, because it terminated fatally. At least, we know not how else these inconsistencies are to be explained.

The details of Dr. Macdowell's cases are meager in the extreme,§ which is the more to be regretted since they are in many points so extraordinary. The account indeed of his first case almost staggers belief, for we are told not only that the patient had no unfavorable symptom, but that when Dr. Macdowell called on the fifth day after the operation he found her making her bed! The particulars of his third case too are very vague; it is stated that the tumour adhered to the left side, but we are not informed how the adhesions were overcome. The ligature which had been placed round the pedicle of the ovary and the Fallopian tube did not come away until the fifth week, after which the patient recovered completely. In his last successful case, operated on in 1817, the patient's life was nearly lost during the operation by the ligature which had been applied round the vessels slipping, and tremendous hemorrhage took place before another could be made secure. The patient recovered from the effects of the operation but continued in bad health.

Mr. Lizars'|| only successful case is to our minds anything but satisfactory. One of the patient's ovaries was removed, and the other was then found to be increased to "nearly the fourth part of the one removed, and was adhering on the right side of the parietes of the pelvis and to the uterus, but comparatively free on the left side." This ovary, however, was left behind; Mr. Lizars desisting at the request of the gentlemen around him. Hemorrhage, which had nearly proved fatal, came on two hours after the operation, and for the succeeding sixty hours her life was in imminent danger. The account of the patient terminates ten weeks after the operation, at which time she was recovering from an inflammatory attack, which had rendered the employment of local depletion necessary; and was "now able again to get out of bed and to take nourishing diet,"

* Mémoires de la Société Royale de Médecine.—Année, 1782, 4to, p. 296.

† Dr. N. Smith of Yale College, Connecticut. His case was originally published in No. xvii. of the American Medical Recorder. A brief notice of it is contained in the London Medical and Physical Journal, vol. xlviii. p. 449; and the paper is found translated into Italian in vol. xxviii. of Omodeis Annali Universali.

‡ Holscher's Annalen, 1841. Heft iii. Seite 261.

§ London Medical Repository, new series, vol. iii. p. 416.

|| Observations on the Extraction of diseased Ovaria, folio.—Edinburgh, 1825, p. 9.

but the ligature had not yet come away. Of the subsequent history of the patient we know nothing.

Dr. A. G. Smith, of Danville, in Kentucky,* operated on a negress, aged thirty, the mother of two children; in whom the first symptom of ovarian disease had appeared two years before. He made an incision from the umbilicus to within an inch of the pubes, but finding the tumour still too large to remove entire he tapped it, when several pints of fluid escaped. The pedicle, which was not larger than the breadth of the broad ligament, was now tied, and the ovary was removed. During the operation the patient made efforts to vomit, and for three days afterwards she suffered from very troublesome nausea and vomiting, which could be controlled only by very large doses of opium. No inflammatory symptom appeared; the ligature came away on the twenty-fifth day, and, with the exception of pains in the loins at the menstrual periods, she continued in good health at the time of the report three years afterwards.

We have never met with the pamphlet of Dr. Quittenbaum, entitled "*Commentatio de Ovarii hypertrophia et historia exstirpationis ovarii hydropici et hypertrophici prospero cum successu factæ,*" but it appears from the statements of Mr. Jeaffreson,† who had seen it, that the operation performed was of the kind recommended by Dr. Clay.

Dr. Rogers,‡ of New York, removed the ovary of a woman twenty years of age, who had been tapped for ascites seven times in the course of two years. After evacuating the ascitic fluid, the enlarged ovary was distinctly perceptible in the left side of the abdomen, and was removed by an incision reaching from two inches above the navel to the pubes. The cyst was firmly adherent to the peritoneum for at least three inches around the umbilicus, but by careful dissection these adhesions were divided. The wound is said to have healed in the course of twenty-eight days without a single untoward symptom having occurred. Beyond this statement no particulars of the patient's progress are given in the paper, and, though promised by Dr. Rogers, have not appeared. Twenty-eight days only had elapsed, from the performance of the operation, when he forwarded the account just quoted to the Gazette.

Dr. Ritter§ operated on a woman aged thirty-one, in whom the disease had existed about a year, and during the course of her fifth pregnancy the increase in the size of the tumour was very rapid. Sixteen weeks after delivery paracentesis abdominis was performed, and twenty-six pounds of fluid were evacuated, and a hard irregular body was then felt extending from the right iliac region to above the umbilicus. A fortnight afterwards this body, which was the enlarged ovary, weighing twelve pounds, was removed by the large incision. During the course of the operation three vessels were tied, each as large as a goose-quill, and adhesions in the epigastric region, at the umbilicus, and in the left iliac region, required to be divided. The patient was very faint during

* London Medical Repository, new series, vol. iii. p. 416, reports the case from the North American Medical and Surgical Journal for January, 1826.

† Transactions of the Provincial Medical Association, vol. v. p. 245.

‡ Medical Gazette, October, 1829.

§ Case reported by Dr. Ehrhart von Ehrenstein in Med. Jahrb. d. K. K. Oester.-Staates, Band xi., 1832, p. 256.

the operation; on the next day she had frequent syncope, shivering, and laborious respiration. On the third day violent febrile symptoms came on, and the patient's life was in imminent danger, until the eighth day, when a discharge of bloody serum from the wound occurred, and was attended with great relief to her symptoms. Her subsequent improvement was slow, but at the end of the ninth week the wound had closed, and her recovery might be regarded as complete.

Dr. Chrysmar,* of Wangen, removed the ovary of a lady thirty-eight years old, who was the mother of seven children. The first signs of the disease had appeared in her seven years before, and for the last three years the increase of the growth had been attended with considerable impairment of her health. The tumour, weighing eight pounds and a half, was removed by an incision extending from the sternum to the pubes. The operation was followed after the lapse of a few hours by slight shivering and hiccup, but no severe febrile symptom occurred at any time. At the end of six weeks she was quite well, and continued so at the date of the report eight years afterwards, having in the interval given birth to another living child.

All must, we think, agree with us in the opinion that the mere announcement that these thirteen persons recovered from the operation would by no means suffice to enable us to estimate it aright. The sufferings endured during its performance, the pains of a protracted convalescence, and the imminent danger in which life was placed in some instances, ought all to be taken into account. The sufferings and the danger too, in these cases, were neither few nor small. Twice (in Mr. Lizars' patient and in one of those operated on by Dr. Macdowell) the patient was exposed to great danger from hemorrhage. Dr. Ritter's patient nearly sank from the shock of the operation, and the violent fever which ensued well-nigh cost her her life. In Dr. Clay's first case, in Dr. A. G. Smith's case, and in that recorded by Mr. Walne, the symptoms were at one time of a very serious nature, though not such as to betoken immediate danger. We are not furnished with details of Dr. Clay's third successful case for the first three days after the operation, and unfortunately we do not know the history of the person whose case is related by Dr. Quittenbaum. In five cases (two of Dr. Macdowell's, one of Dr. Clay's, Dr. Rogers's, and Dr. Chrysmar's) the patients went on favorably from the very beginning; but in Macdowell's and Rogers's cases there is a most blameworthy deficiency of information on all points relating to the patient's progress. In six of the thirteen cases then, there is evidence of the life of the patient having been placed in some degree of jeopardy, while in three only of those in which the patients are alleged to have recovered without any bad symptom, are we furnished with such details as we have a right to require. Moreover, Dr. Macdowell's first case, that of Dr. Chrysmar and of Dr. A. G. Smith are the only ones in which a length of time had passed since the operation, sufficient to test the permanence of the cure or to show that the other ovary did not become the subject of disease. In Mr. Lizars' patient it is expressly stated that at the time of the operation both ovaries were diseased though only one was extirpated, and we have Dieffenbach's authority for the opinion that this would be the case

* Case reported by Dr. Hopfer, in Graefe und Walther's Journal, Band xii. Seite 60.

in many instances. The appearance of the solid part of the ovary in the two successful cases by Dr. Clay, in which he has described the tumour, would lead to the apprehension that the disease in both instances partook of a malignant character, and that consequently no lasting benefit will result from the operation.

There are objections, however, to the operations far more conclusive than any which can be deduced from the inadequate nature of the testimony in its favour. Not only did six of the persons, whose ovaries were extirpated, die from the effects of the operation, but in eight instances, after the abdominal cavity had been laid open, the removal of the tumour was found impracticable, and the lives of three of these patients were sacrificed to the fruitless and ill-judged interference of the surgeon.

We will now proceed to an analysis of these melancholy cases, tedious though it may be to our readers, for we feel it to be a sacred duty when erroneous statements have been made on a subject so deeply interesting, to expose their fallacy at any cost of time or labour.

We will first examine those six cases in which the extirpation of the ovary was effected at the expense of the patient's life; and the fearful character of the operation will be obvious from the fact that these six cases give an average duration of life after its performance of only forty-five hours.

In Dr. Macdowell's fifth case, operated on in the year 1818, the tumour adhered by long and slender attachments to several of the adjacent parts, and on being divided several of them bled, and required to be secured by ligatures. Violent peritonitis came on, and the patient died on the third day after the operation, having suffered from severe pain in the abdomen and obstinate vomiting. The peritoneum was found violently and extensively inflamed.

In Professor Lizars' third case, the tumour adhered so strongly to the parietes of the abdomen, to the colon, and to the brim of the pelvis, that the operator despaired of being able to detach it, but at length succeeded by dissecting and occasionally tearing the adhesions with caution. From the moment when the operation was completed the patient's symptoms were alarming in character and grew more serious until her death, which took place fifty-three hours afterwards. Of this patient Dr. Clay observes that she "had previously suffered from an attack of cholera, which, with equal probability with the operation, might be considered as the cause of death." We hardly know how to designate this assertion of Dr. Clay's. The patient, indeed, is stated by Mr. Lizars to have been very dangerously ill from cholera when he first saw her, on the 13th of March, but a few days after he found her "at the fireside, cheerful and having more strength than any one could have anticipated;" and the operation was not performed until March 22, at which time there do not appear to have been any remains of her former indisposition. To remove all doubt from the minds of our readers, however, we will transcribe the account given by Mr. Lizars of the appearances found on examination of the body, all allusion to which Dr. Clay suppresses:

"On cutting the stitches of the wound," says Mr. Lizars, "it was found adhering in some points, particularly at the upper or sternal part; at the lower part a little serum and purulent matter issued out; and on reflecting back the parietes of the abdomen, the omentum and intestines were found adhering to the neighbourhood of the wound. The peritoneum investing the parietes, which adhered to the tumour, and also those portions of this membrane investing the colon and small intestines which adhered to the tumour, were of a blueish black appearance, and tore with ease under the fingers, being evidently gangrenous. Small patches of inflammation were observable in other parts of the intestines, particularly the colon. In the pelvic cavity there were a few ounces of serous fluid, with flakes of coagulable lymph floating in it. The pedicle was then seen to be the broad ligament of the uterus, and the uterus itself was found perfectly healthy; the other ovarium was of its natural size, had a coating of coagulable lymph, but was tolerably firm when bisected; the fallopian tube was turgid and red in colour."

We would hope for Dr. Clay's sake that he had not been at the trouble of reading the account of the post-mortem examination when he made the statement we have quoted.

Dr. Chrysmar's first case was that of a person aged forty-seven, in whom disease of the ovaries had existed ever since the cessation of her menses four years previously. Her abdomen had for a year attained as large a size as that of a woman in the ninth month of pregnancy, and her health had become very greatly impaired. Ascites was known to coexist with the ovarian disease, but paracentesis had never been performed. On opening the abdomen, five quarts of greenish-yellow fluid escaped. The tumour was closely adherent to the transverse colon and to the great arch of the stomach, and when removed was ascertained to weigh 7⅓lbs. Its removal was followed by great anxiety and frequent vomiting which nothing could quell; the powers of the patient sank hourly, and she died thirty-six hours after the operation. On the post-mortem examination peritonitis was found, with effusion of lymph and pus into the abdomen, and adhesion of the intestines to each other. The peritoneum in the whole neighbourhood of the ovary was gangrenous, and gangrenous patches were found on the colon and stomach at the places where the adhesions had existed.

The patient in Dr. Chrysmar's third case was thirty-eight years old. Her health had been habitually indifferent, and for eight years she had suffered from anasarca with ascites and enlarged liver, and for at least as long a period there had existed signs of enlargement of the left ovary. Paracentesis of the abdomen had been performed once when eight quarts of fluid were let out, but without any amendment of her condition. On opening the abdomen three quarts of yellowish fluid were let out, and the tumour was then found to be adherent only towards the promontory of the sacrum, and its pedicle was only four inches wide. Its removal, however, was followed almost immediately by syncope, which returned frequently, and the patient died in thirty-six hours after the performance of the operation. A post-mortem examination disclosed traces of peritonitis with purulent effusion into the abdominal cavity, and gangrenous patches on the peritoneum.

Subsequently to the publication of Dr. Clay's pamphlet, Dr. Granville sent a brief notice to the Medical Gazette (Jan. 13, 1843,) of a case in which he removed a diseased ovarium, but in which the patient died three days afterwards. Dr. Granville asserts, but furnishes no kind of proof, that her death was caused by her having been over depleted by her medical attendant. Be this as it may, however, it will probably be allowed that it is but a fair inference to assume that symptoms of peritonitis existed, sufficiently severe to render the employment of some degree of depletion necessary.

Lastly, we may mention Dr. Clay's fifth case, in which the patient all but died on the operating table, and will now pass to those instances in which the operation was from various causes left incomplete.

Shortly after his first successful operation, Dr. Macdowell* attempted it a second time, but found the ovary so firmly connected with the bladder and the fundus of the uterus, that its extirpation was impossible. He therefore merely made an incision into the ovary, from which a considerable quantity of gelatinous fluid and blood escaped, and closed the wound, but not until more than a quart of blood had escaped into the abdominal cavity. This was removed before the wound was closed, the patient recovered and returned to her usual occupations. No information is given as to the symptoms which attended the patients convalescence, but we learn that at the end of six years the growth had regained its former size.

In the first case on which Mr. Lizars operated, no tumour existed; the life of the patient was jeoparded to no purpose, and for some days afterwards the symptoms were extremely alarming; yet because life was not actually lost by the operation, Dr. Clay appeals to it as an instance of its successful performance.

In Mr. Lizars' fourth case, the blood-vessels of the omentum were found to be enormously enlarged, and running on the surface and into the substance of the tumour, which accordingly was thought most prudent not to meddle with. Being anxious, however, to reduce the bulk of the tumour, Mr. Lizars punctured it at the lower part, but only pure blood flowed.

We again refer to Mr. Lizars' own account of the symptoms which followed the operation. The patient's sufferings were severe, but she appears to have escaped with her life, though her history is not carried beyond a fortnight after the operation, at which time she had not left her bed, and the wound though healing was not healed.

This too is one of Dr. Clay's successful cases, and not a hint is given by him that in this or in any other instance, (except in that of Mrs. Dillon operated upon by himself,) the attempt to remove a diseased ovarium has failed from the extent of adhesions. And yet Dr. Clay has read Mr. Lizars' book, and makes quotations from it when they serve his purpose! On such want of good faith we forbear to comment.

Dr. Granville† attempted the operation in the year 1826, but desisted after having laid bare the tumour, on account of the number and extent of the adhesions. The patient recovered rapidly from the effects of the operation. One point mentioned in the details of the case is important as illustrating a grand objection to the operation. It is stated that "Dr. Granville passed his hand into the abdominal cavity, and ascertained that although the tumours felt loose when examined externally, they were in fact adhering in various directions by strong bands."

Professor Dieffenbach‡ attempted the removal of an ovarian tumour in the case of a Polish lady, forty years of age, in whom the disease had been the slow growth of ten or twelve years. Considerable difference of opinion as to its nature existed among the medical practitioners who saw

* This case is one enumerated as successful by Dr. Clay!

† London Medical and Physical Journal, vol. lvi. p. 141.

‡ Rust's Magazin. Band xxv. Seite 339. (Jahrgang 1828.)

the lady, but the extreme mobility of the tumour and the excellent health of the patient induced Professor Dieffenbach to attempt the operation. It was found, however, after laying open the abdominal cavity that the tumour was connected with the vertebral column, that its pedicle was very broad, and contained vessels which pulsated with great force. A puncture made into the tumour was followed by hemorrhage which it required compression to arrest. The extirpation of the growth being deemed hazardous, the wound was closed and a bandage applied. Violent pain, hiccough, and vomiting, all the most aggravated symptoms in short of a strangulation of an intestine came on immediately; the abdomen became tense and tumid, and the size of the tumour was evidently increased. These symptoms yielded after the employment for some days of a strict antiphlogistic treatment, but were followed by great debility, accompanied with a yellow tinge of the skin and a rapid and hurried pulse. By degrees, however, as the discharge from the wound diminished, these symptoms subsided and the patient ultimately recovered her health.

In three instances, one of which is the first fatal case related by Dr. Clay, the attempt to remove the tumour was not merely unsuccessful, but occasioned the death of the patient. A second case occurred to Dr. Martini of Lübeck, (Rust's Magazin, xv. Band, Seite 436,) who attempted the removal of a diseased ovary from a person twenty-four years old, in whom the first signs of disease had appeared fifteen months previously, three months after she had given birth to a dead child. Paracentesis was performed four times, and after the fourth puncture, an attempt was made to excite adhesions between the walls of the cyst, by injecting an irritating fluid into its cavity, but without success. The attempt was repeated after a short time, but on making a puncture with this view, no fluid escaped, and it was inferred that the contents of the tumour had become solid. Dr. Martini now resolved to attempt its extirpation, but after opening the abdominal cavity he ascertained that the sac which formed the upper part of the tumour was connected inferiorly with a firm growth of a cartilaginous hardness which was so firmly adherent to the bladder, rectum, and walls of the pelvis, that it could not be detached. The sac when punctured gave exit to three pints of fluid, and Dr. Martini was forced to content himself with cutting off the sac from the solid tumour with which it was connected. The patient fainted during the operation; on the following day, however, she had rallied, but on the morning of the third day, her abdomen became distended, she began to sink, hiccup came on, she fell into a state of syncope and died fifty-six hours after the operation. There was a considerable quantity of bloody fluid in the abdomen, and a great exudation of lymph immediately under the incision through the abdominal parietes; but the intestines generally were pallid. The hard tumour in the pelvis which was formed by the left ovary, had become soft since the performance of the operation, and now contained cavities full of pus, and a steatomatous matter.

An anonymous writer* details the particulars of a case in which an attempt was made to remove the ovary of a woman, forty-eight years old, in whom the first symptoms of disease had appeared a year before.

* Froriep's Notizen. Band xiv. Numero 300. Seite 215.

After the tumour had existed six months paracentesis was performed, and in the course of the ensuing half year was repeated five times. On attempting the extirpation of the cyst it was found not to be seated on a narrow pedicle, but to have a broad base, and to be firmly connected with the os innominatum. Nothing more therefore could be done than to remove the upper two thirds of the sac, the walls of which were from two thirds of an inch to an inch in thickness, and the fluid it contained was extremely thick and gelatinous. The subsequent history of the patient is detailed very briefly, but it is stated that fever and tetanic symptoms came on, of which the patient died on the sixth day after the operation. The peritoneum and intestines generally were uninflamed, but the omentum was inflamed, and the remains of the sac formed a cavity which was filled with purulent matter.

It was our intention to have examined the comparative merits of other operative proceedings which have been at different times adopted for the cure of ovarian dropsy, but we have already exceeded our limits. The erroneous statements, however, by which Dr. Clay endeavours to bring the operation practised by Mr. Jeaffreson into disrepute are so remarkable that we cannot pass them by unnoticed. Not content with omitting all mention of the successful cases recorded by Mr. Gorham in the Lancet for October 1839, he asserts that Dr. Dohlhoff operated on three persons by the small incision, and that of these three two died, and in the third who recovered no tumour was found. Now we deny that in any of these three cases the operation was at all similar to that practised by Mr. Jeaffreson.* In the first patient the incision was indeed originally only two inches long, and the tumour was punctured by a trocar. Its contents were found too viscid to flow through the canula; the incision was then very *considerably enlarged*, and the tumour laid open sufficiently for the operator to bale out its contents with a cup. After its bulk had been thus reduced, the tumour was removed. This patient died sixteen hours after the operation, and peritonitis with effusion were found on a post-mortem examination.

The incision in the second case was sufficiently large for the bystanders to perceive that the vessels of the omentum were morbidly dilated, that small white tumours of the size of a bean covered the surface both of the omentum and the peritoneum lining the abdominal walls. It likewise allowed them to see that the tumour had very intimate adhesions to deep-seated organs, on which account the attempt to extirpate it was discontinued, but eight hours afterwards the patient died.

In the third case we are informed that after the abdominal cavity had been laid open, and the hand had been introduced into it in search of a tumour, none could be found; [nach eröffneter Unterleibshöhle und nach dem Eingehen mit der Hand in dieselbe fand sich keine Spur einer Geschwulstvor.] This person continued in a dangerous condition for some days, but ultimately recovered.

Now, on what principle these cases are classed among those operated on in the method practised by Mr. Jeaffreson we are perfectly at a loss

* See the particulars of these cases in Rust's Magazin, Bd. li. Jahrg. 1838, S. 77; or in Kleinert's Repertorium, 1838, Heft vi.

to conceive; for to our minds they far more closely resemble cases operated on by the large incision.

It is not, however, our object to advocate the operation by the small incision, for our estimate of any such proceeding coincides exactly with that expressed by the late Dr. Hamilton, in his Practical Observations on Midwifery. We have merely sought to exhibit the real amount of danger which attends *one* of the operations that have been practised for the cure of ovarian dropsy, *one* which its advocate describes as yielding results far more favorable than those of amputation. Our readers may judge for themselves how far this statement is consonant with truth—to our thinking the facts need no comment. We earnestly hope that they will prevent the younger members of the profession from being dazzled by the *alleged* success of an operation, which though it may excite the astonishment of the vulgar, calls neither for the knowledge of the anatomist nor the skill of the surgeon. In some continental universities the candidates for the doctor's degree takes an oath "Nullius unquam hominis vitam ancipiti tentaturum experimento;" a fundamental principle of medical morality which we conceive is outraged whenever an operation so fearful in its nature, often so immediately fatal in its results, as gastrotomy, is performed for the removal of a disease, of the very existence of which the surgeon is not always sure; of the curability of which, by his interference, he must be in the highest degree uncertain. There are unfortunately no means of gaining notoriety in the practice of medicine so speedily as by the performance of some bold operation, which the aged and the timorous are reluctant to attempt, and we can fully sympathise with those who, just setting out in their medical career, are unwilling to let slip any opportunity of advancing their reputation. We would, however, entreat all such to ponder well before they jeopardy the lives of their patients by operations such as that the claims of which we have been investigating, and we would say to each one, in the words of Hufeland "Thine is a high and holy office, see that thou exercise it purely, not for thine own advancement, not for thine own honour, but for the glory of God, and the good of thy neighbour. Hereafter thou wilt have to give an account of it."*

* Since the preceding pages were written, Mr. Walne has performed a second successful operation; the announcement of which appears in the Medical Gazette for July 7. He has favoured us with the following particulars of the case: "The patient experienced an attack of inflammation of the iliac vein, producing enlargement of the inferior extremity; in short a complete phlegmasia dolens of that side on which the tumour was attached to the uterus, and of course the pedicle tied in the operation. Yet she has recovered, except being still feeble from this attack."

